# Supplementary material for: Genome-scale metabolic modelling when changes in environmental conditions affect biomass composition
Source: PLoS Comput Biol. 2021 May 24;17(5):e1008528. doi: 10.1371/journal.pcbi.1008528 (PMC8177628; doi:10.1371/journal.pcbi.1008528)
Supplement: S1 Text — This supporting information text contains the numbers for single-gene knockout results (Table A in S1 Text), presents non-relative heatmaps (cf. Fig 3; Fig A in S1 Text) and provides a visual mapping of environmental space coordinates used in the single-gene knockout analysis (Fig B in S1 Text). In Fig C in S1 Text, we show the gene essentiality similarity of the model predictions in relation to experimental data by Rousset et al. [37], the raw data is shown in S2 Table. (PDF) [file pcbi.1008528.s001.pdf]

Supporting information S1 Text - Overview

Table A in S1 Text
Table of knockouts.

Fig A in S1 Text
Direct plots for Fig 3 in the manuscript.

Fig B in S1 Text
Selected points for single-gene knockout analysis presented in Fig 5.

Fig C in S1 Text
Gene essentiality similarity in *E. coli* K12 MG1655 in comparison with the data by Rousset et al.

Table A in S1 Text
Table of knockouts.

Single gene knockouts at 10 different environments using FBA with the biomass functions being UL, NL, or CL, and for the methods BTW, HIP and HIP-I. The environments are defined by their carbon and nitrogen uptake fluxes, where the flux value represents the maximum uptake limit. Number of genes are reported according to induced mutant growth phenotype relative to the wild type in the following intervals: $[0, 0.01)$ / $[0.01, 0.50]$ / $(0.50, 0.88]$ / $(0.88, 0.98]$ / $(0.98, 1]$.

(C,N) max flux	UL	NL	CL	BTW	HIP	HIP-I
1 : (18, 8.5)	250/0/8/0/1258	250/8/2/35/1221	250/0/0/0/1266	250/0/0/10/1256	250/0/8/0/1258	250/8/0/0/1258
2 : (15.29, 4.28)	241/0/0/0/1275	250/8/2/35/1221	241/0/0/0/1275	250/0/8/20/1238	250/0/8/0/1258	250/8/0/0/1258
3 : (15.16, 6)	250/0/8/0/1258	250/8/2/35/1221	241/0/0/0/1275	250/0/0/10/1256	250/0/8/0/1258	250/8/0/0/1258
4 : (13.5, 1.5)	241/0/0/0/1275	240/0/8/0/1268	240/0/0/0/1276	233/0/0/8/1275	240/0/8/0/1268	250/0/8/0/1258
5 : (12.91, 2.5)	241/0/0/0/1275	250/8/0/0/1258	241/0/0/0/1275	250/0/8/0/1258	250/0/8/0/1258	250/0/8/0/1258
6 : (12.32, 3.5)	241/0/0/0/1275	250/8/2/35/1221	241/0/0/0/1275	250/0/8/20/1238	250/0/8/0/1258	250/8/0/0/1258
7 : (11.25, 5.3)	250/0/8/0/1258	250/8/2/35/1221	241/0/0/0/1275	250/0/0/10/1256	239/0/8/0/1269	250/8/0/0/1258
8 : (9.74, 1.24)	241/0/0/0/1275	240/0/8/0/1268	241/0/0/0/1275	233/0/0/8/1275	240/0/0/0/1276	250/0/0/0/1266
9 : (6.91, 2.09)	241/0/0/8/1267	240/8/2/35/1231	241/0/0/0/1275	240/0/8/20/1248	240/0/8/0/1268	250/0/8/0/1258
10 : (1.5, 0.68)	247/0/0/0/1269	245/0/19/18/1234	249/0/0/0/1267	235/0/0/19/1262	249/0/0/0/1267	258/0/0/0/1258

Fig A in S1 Text

Direct plots for Fig 3 in the manuscript.

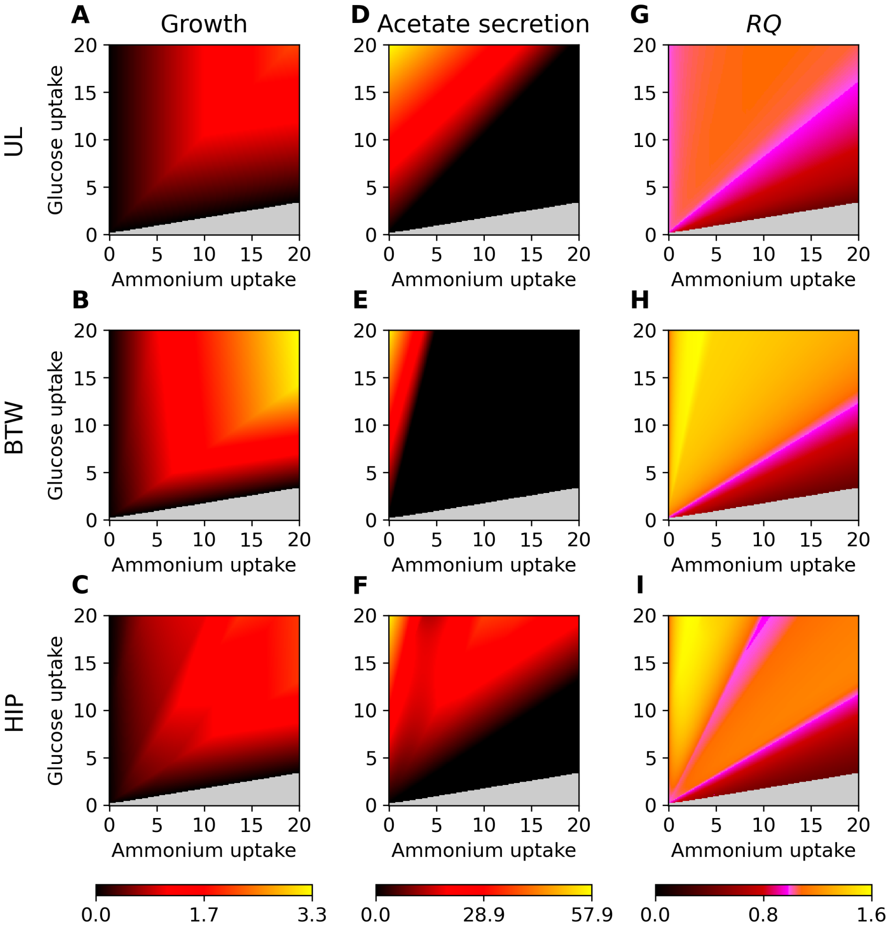

Note especially the light-grey zero-areas in the direct comparison. Note further that the growth rates are in h^{-1} $gCDW^{-1}$, the acetate secretion in

S1A Fig The plot shows the non-relative heatmaps presented in Fig 3, for BTW, HIP and UL for a direct comparison. Note especially the light-grey zero-areas in the direct comparison. Note further that the growth rates are in h^{-1} $gCDW^{-1}$, the acetate secretion in mmol $gCDW^{-1}$ h^{-1}, and RQ is unit less.

Fig B in S1 Text

Selected points for single-gene knockout analysis presented in Fig 5.

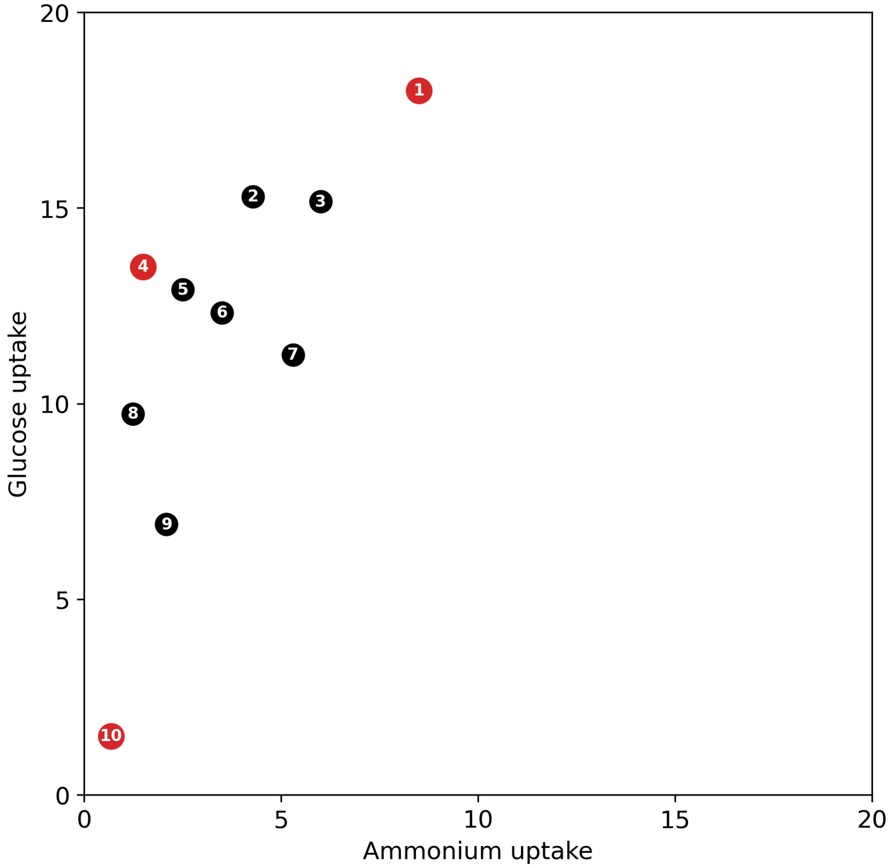

S1B Fig The figure shows the glucose-ammonium environmental space and the three BOFs UL, NL, and CL (marked in red). The other points marked in the figure are the coordinate locations for the single gene knockout analysis. The numbering of the points corresponds to the numbering in Fig 5.

Fig C in S1 Text

Gene essentiality similarity in *E. coli* K12 MG1655 in comparison with the data by Rousset et al.. The data shows, that the M9 minimal medium has the highest similarity to the predicted gene essentiality, whereas both rich media score comparable. Note that the LB medium seems to be consistently closer to the computational predictions. The NL BOF generates the highest similarity to experimental data, the CL the lowest. Further, the CL BOF shows the lowest change in gene essentiality across the given environmental coordinates. In summary, we find that the gene essentiality depends on the BOF and the environment in both, experimental data and *in silico* predictions. Note that the experimental data for M9 medium is generated with glucose in exponential (unlimited) growth, hence for one uptake coordinate. The data this figure is based on can be found in S2 Table.

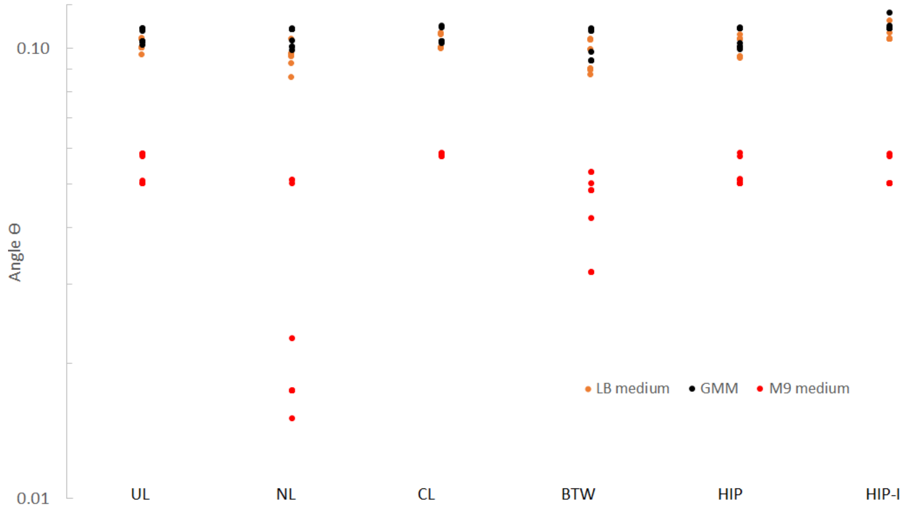

S1C Fig The figure shows the similarity of the gene essentiality for the various methods / biomasses generated for each environmental coordinate in comparison to the experimental data.
